# Supplementary material for: The genome sequence of a picture-winged fly, Melieria crassipennis (Fabricius, 1794)
Source: Wellcome Open Res. 2024 Oct 18;9:614. [Version 1] doi: 10.12688/wellcomeopenres.23224.1 (PMC11569385; doi:10.12688/wellcomeopenres.23224.1)
Supplement: Peer review history

# Reviewer report: Reviewer response for version 1

**DOI:** 10.21956/wellcomeopenres.25580.r109168

**Published:** 2024-11-21

**Authors:** Phillip Shults, Edward Bird

**Affiliations:** 1 USDA-ARS, Manhattan, USA; 2 Department of Entomology, Kansas State University, Manhattan, Kansas, USA

This is a genome report for the picture wing fly *Melieria crassipennis*. The article contains a detailed explanation of who the sample was processed and sequenced as well as the bioinformatic pipelines used for the genome assembly. All the methods seem appropriate, and all the reported results are informative. This is well written and concise. Below are a few comments and suggestions to improve the manuscript.

**General Comments:**

 Sample Extraction Methods

 Linked Protolcol.io is a repository of protocols. It is difficult to tell exactly which version of sub protocol was used. I would like to see each individual protocol cited for each step.

 “While not fully phased, the assembly deposited is of one haplotype. Contigs corresponding to the second haplotype have also been deposited.”

 This doesn’t make sense to me. “Not fully phased” means that it isn’t haplotype resolved, however they go on to say it is all from one haplotype?

 They ran HiFiASM in primary mode, which does not result in a phased assembly, but rather a primary and alternative assembly. While this does result in long phased stretches, the entire genome is not phased, or haplotype resolved.

 Assembly Screen for Cobionts and Contaminants (ASCC) pipeline

 The article is in prep, but there is no description anywhere of what/how this does. Is not in this either https://gitlab.com/wtsi-grit/rapid-curation . This only covers TreeVal and PretextView

**Small Comments:**

 The abstract has a type “(tArthropoda;” Just double check for other small errors.

 This is minor and purely my opinion, I would like to see what the authors think

 “…homology is unreliable for sex chromosome identification in Diptera due to frequent sex chromosome turnover ( Vicoso & Bachtrog, 2015).” I think there is a better argument to be made here. The authors did not look at the sex chromosomes because there was no male assembly is a completely valid reason by itself, but I think having no studies of sex determination in Ulidiidae is an important point. Yes, sex determination in flies can be complicated, but many Brachycera have heteromorphic XY sex chromosomes so this system could not be complex. We don’t really know and so this statement is a bit superficial. Of course, the reason those sex determination studies would be needed for this family is because of the potential complexity so maybe there’s a way to say both?

 “It aligns the PacBio reads in SAMtools and minimap2 ( Li, 2018 ) and generates coverage tracks for regions of fixed size.”

 Samtools isn’t a read aligner, but rather is for Reading/writing/editing/indexing/viewing SAM/BAM/CRAM

 In YaHS - What does –bread do? It’s not anywhere in YaHS documentation

Are sufficient details of methods and materials provided to allow replication by others?

Yes

Is the rationale for creating the dataset(s) clearly described?

Yes

Are the datasets clearly presented in a useable and accessible format?

Yes

Are the protocols appropriate and is the work technically sound?

Yes

Reviewer Expertise:

I study fly genomics.

We confirm that we have read this submission and believe that we have an appropriate level of expertise to confirm that it is of an acceptable scientific standard.

# Reviewer report: Reviewer response for version 1

**DOI:** 10.21956/wellcomeopenres.25580.r109162

**Published:** 2024-11-16

**Authors:** Charles Plessy

**Affiliations:** 1 Okinawa Institute of Science and Technology, Okinawa, Japan

This report follows the standard template of other Darwin Tree of Life Consortium data notes. As I am not a fly biologist I have focused on the quality assessment of the genome assembly. The completeness (BUSCO score) is good. The Hi-C contact map does not show obvious signs of scaffolding errors. The size of the alternate assembly is comparable to the one of the primary pseudo-haplotype. In addition, I have aligned the chromosome-level pseudo-haplotype assembly to publicly available chromosome assemblies in the same genus for *M. omissa* and *M. picta*. Alignment to *M. omissa *shows a 1-to-1 syntenic relation between pairs of chromosome-scale scaffolds, which is a good sign.

Are sufficient details of methods and materials provided to allow replication by others?

Yes

Is the rationale for creating the dataset(s) clearly described?

Yes

Are the datasets clearly presented in a useable and accessible format?

Yes

Are the protocols appropriate and is the work technically sound?

Yes

Reviewer Expertise:

Comparative genomics

I confirm that I have read this submission and believe that I have an appropriate level of expertise to confirm that it is of an acceptable scientific standard.

# Reviewer report: Reviewer response for version 1

**DOI:** 10.21956/wellcomeopenres.25580.r109169

**Published:** 2024-11-13

**Authors:** Anthony Bayega

**Affiliations:** 1 McGill University, Montreal, Quebec, Canada

Mitchell et al. provide an overview description of the genome of *Melieria crassipennis* which they sequenced and assembled. I commend them for their efforts. My comments follow below:

 1. In Figure 3, the label on the Y-axis which reads “ERR102…” could be changed to something more informative to help the reader make sense of this figure.

 Overall, the authors provide a high-quality genome and also assign much of it to chromosomes. Although much work remains to order the scaffolds and complete the gaps and also structurally and functionally annotate the genome, the current work will indeed be valuable to the whole community. I therefore recommend the indexing of this genome so that this resource becomes widely accessible to the scientific community.

Are sufficient details of methods and materials provided to allow replication by others?

Yes

Is the rationale for creating the dataset(s) clearly described?

Yes

Are the datasets clearly presented in a useable and accessible format?

Yes

Are the protocols appropriate and is the work technically sound?

Yes

Reviewer Expertise:

Genomics

I confirm that I have read this submission and believe that I have an appropriate level of expertise to confirm that it is of an acceptable scientific standard.
